# Poor performance of quick-SOFA (qSOFA) score in predicting severe sepsis and mortality – a prospective study of patients admitted with infection to the emergency department

**DOI:** 10.1186/s13049-017-0399-4

**Published:** 2017-06-09

**Authors:** Åsa Askim, Florentin Moser, Lise T. Gustad, Helga Stene, Maren Gundersen, Bjørn Olav Åsvold, Jostein Dale, Lars Petter Bjørnsen, Jan Kristian Damås, Erik Solligård

**Affiliations:** 1 Clinic of Anesthesia and Intensive Care, St Olav University Hospital, Trondheim, Norway; 2 Clinic of Emergency Medicine and Prehospital Services, St Olav University Hospital, Trondheim, Norway; 3 Department of Circulation and Medical Imaging, NTNU, Norwegian University of Science and Technology, Po box 8905, N-7491 Trondheim, Norway; 4 Department of Endocrinology, St Olav University Hospital, Trondheim, Norway; 5 Department of Infectious Diseases, St Olav University Hospital, Trondheim, Norway; 6 Department of Public Health and Nursing, NTNU, Norwegian University of Science and Technology, Trondheim, Norway; 7 Centre of Molecular Inflammation Research of Cancer Research and Molecular Medicine, NTNU, Norwegian University of Science and Technology, Trondheim, Norway; 8 Department of Medicine, Levanger Hospital, Nord-Trøndelag Hospital Trust, Levanger, Norway; 9 Mid- Norway Sepsis Research Center, NTNU, Norwegian University of Science and Technology, Trondheim, Norway; 10 Faculty of Medicine, NTNU, Norwegian University of Science and Technology, Trondheim, Norway

**Keywords:** Sepsis, Emergency Department (ED), Prospective, quick-SOFA (q-sofa), Systemic inflammatory response syndrome (SIRS), Rapid emergency triage and treatment system (RETTS)

## Abstract

**Background:**

We aimed to evaluate the clinical usefulness of qSOFA as a risk stratification tool for patients admitted with infection compared to traditional SIRS criteria or our triage system; the Rapid Emergency Triage and Treatment System (RETTS).

**Methods:**

The study was an observational cohort study performed at one Emergency Department (ED) in an urban university teaching hospital in Norway, with approximately 20,000 visits per year. All patients >16 years presenting with symptoms or clinical signs suggesting an infection (*n* = 1535) were prospectively included in the study from January 1 to December 31, 2012. At arrival in the ED, vital signs were recorded and all patients were triaged according to RETTS vital signs, presenting infection, and sepsis symptoms. These admission data were also used to calculate qSOFA and SIRS. Treatment outcome was later retrieved from the patients’ electronic records (EPR) and mortality data from the Norwegian population registry.

**Results:**

Of the 1535 admitted patients, 108 (7.0%) fulfilled the Sepsis2 criteria for severe sepsis. The qSOFA score ≥2 identified only 33 (sensitivity 0.32, specificity 0.98) of the patients with severe sepsis, whilst the RETTS-alert ≥ orange identified 92 patients (sensitivity 0.85, specificity 0.55). Twenty-six patients died within 7 days of admission; four (15.4%) of them had a qSOFA ≥2, and 16 (61.5%) had RETTS ≥ orange alert. Of the 68 patients that died within 30 days, only eight (11.9%) scored ≥2 on the qSOFA, and 45 (66.1%) had a RETTS ≥ orange alert.

**Discussion:**

In order to achieve timely treatment for sepsis, a sensitive screening tool is more important than a specific one. Our study is the fourth study were qSOFA finds few of the sepsis cases in prehospital or at arrival to the ED. We add information on the RETTS triage system, the two highest acuity levels together had a high sensitivity (85%) for identifying sepsis at arrival to the ED - and thus, RETTS should not be replaced by qSOFA as a screening and trigger tool for sepsis at arrival.

**Conclusion:**

In this observational cohort study, qSOFA failed to identify two thirds of the patients admitted to an ED with severe sepsis. Further, qSOFA failed to be a risk stratification tool as the sensitivity to predict 7-day and 30-day mortality was low. The sensitivity was poorer than the other warning scores already in use at the study site, RETTS-triage and the SIRS criteria.

**Electronic supplementary material:**

The online version of this article (doi:10.1186/s13049-017-0399-4) contains supplementary material, which is available to authorized users.

## Background

The quick Sequential related Organ Failure Assessment (qSOFA score) was this year proposed as a risk stratification tool that is more specific than the Systemic Inflammatory Response Syndrome (SIRS) criteria in order to urge the assessment of organ failure, initiate or escalate appropriate sepsis therapy, refer patients to the Intensive Care Unit (ICU) and to help identify life threatening infection [1, 2]. However, the qSOFA recommendation was formed from retrospective analysis of a database and the critique of its clinical usefulness soon emerged [3, 4]. The third task force strongly recommended international validation in different study settings [2]. The first validation studies of suspected infection patients outside the ICU found that the SIRS and National Early Warning Score (NEWS) and the Modified Early Warning Score (MEWS), both commonly used scores in the UK, were more accurate than the qSOFA [5, 6]. A recent study found that the patients worst qSOFA score during upon arrival to an Emergency Department (ED) performed better than the SIRS criteria, however prospective studies are still needed to assess if qSOFA can be used as a screening tool at arrival [7]. Our aim was to evaluate the clinical value of the qSOFA score as a screening tool for sepsis in patients at time of arrival with infection to an Emergency Department (ED). We examined the clinical usefulness of qSOFA to predict severe sepsis and seven- and 30-day mortality and compared its performance to the SIRS criteria and the Rapid Emergency Triage and Treatment System (RETTS), which is commonly used for deciding patients’ acuity level at arrival to an ED [8].

## Methods

### Study group

The study was performed in the ED at St. Olav’s Hospital, an urban university teaching hospital with 700 beds in Norway serving as a local hospital for 280,000 inhabitants and as referral hospital for 700,000 inhabitants. The main ED receives all patients older than 16 years of age, but patients in all age groups who present with multiple traumas, haemodynamic instability, or a need for advanced life support (ALS) interventions are also seen here. Obstetric and gynaecologic; ear-, nose-, and throat (ENT); and paediatric patients are typically seen 24/7 at separate EDs or outpatient clinics within the hospital. EDs in Norway are not designed to provide primary health care. Only patients referred by a general practioner (GP) on call, or another physician, can be admitted to the ED, except for patients who are transported directly from the scene by emergency medical services (EMS). The ED has approximately 20,000 hospital visits per year. The admission rate in this study period to intra-hospital care was 90%. We prospectively included all patients ≥16 years of age with a new onset of suspected or confirmed infection according to the Emergency Symptoms and Signs algorithm for infection (ESS47) from January 1 to December 31, 2012 (new onset defined if no previous hospitalization for infection last 30 days). Thirty patients left the ED before registration or had no identification and were excluded from the study. We included patients who met the criteria for the 4 highest acuity levels according to the Rapid Emergency Triage and Treatment System (RETTS) [8]. All patients were triaged by a nurse and then assessed by an intern. Patients with blue triage were not included in the study as this category is usually referred to treatment in a care facility without all the resources available in the ED [9].

### Variables

#### Clinical data, triage categories and laboratory measurements

All clinical data were measured upon arrival to the ED, the following was measured and registered in the ED database (Akuttdatabasen, version 1.5.5); the presenting complaints according to ESS47 and vital signs like respiratory rate (RR, per minute), SpO_2_ (%), heart rate (HR, per minute), temperature (measured in ear, °C), systolic blood pressure (SBP, mmHg) and Glasgow coma scale (GCS).

The acuity level was given promptly in the ED from highest to lowest priority level; red (physician immediately), orange (physician within 20 min), yellow (can wait), or green (must wait). The triage categories are based on the patients’ most urgent presenting symptom according to the ESS47-criteria for infection and seriousness of deviations from normal vital signs. Red triage (RETTS-R) was given if the patient had petechiae or if one vital sign was observed within following criteria; obstructed airway, stridor, RR >30 or <8, SpO2 < 90% with supplemental oxygen, HR > 130 if sinus rhythm or >150 otherwise, SBP < 90 mmHg, unconscious/ GCS < 9 or cramps. Orange triage was given if the patient was on immunosuppressive medication, had previous surgery with use of prosthetic parts, had fever >38°C with shivering or if vital signs met one of these criteria; temperature >41 °C or <35 °C, RR > 25, SpO2 < 90% without supplemental oxygen, HR > 120 or <40, somnolent/GCS 9-14. Yellow triage was defined if there were signs of a serious local infection or if one of these vital signs were measured within these limits; SpO_2_ 90-95% without oxygen, HR > 110 or <50, acute disorientation or temperature >38 °C. Patients with green priority have vital signs close to normal range and less urgent complaints than yellow, orange and red patients [9].

#### Definition of SIRS, sepsis, severe sepsis and quick-SOFA (qSOFA)

All the following definitions were calculated based on the clinical measurements upon ED arrival. Sepsis was defined as documented or suspected infection and ≥2 signs of SIRS: temperature > 38.0 or <36.0 °C, HR > 90/min, RR >20/min or PaCO_2_ < 4.3 kPa, white blood cell count >12.0 x 10^9^/l or <4.0 x 10 ^9^/l [10]. We also included an analysis with SIRS criteria without leukocytes to evaluate if this SIRS without the wait for laboratory results had equal sensitivity and specificity as SIRS criteria with leucocytes.

**Fig. 1:**
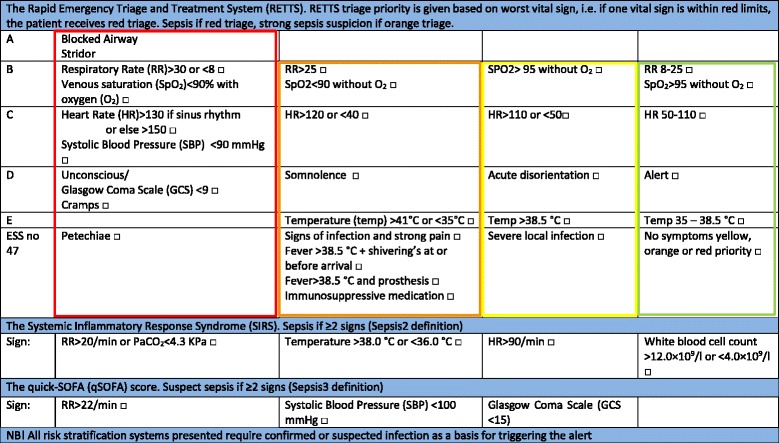
Overview of the different risk stratification tools for sepsis

Severe sepsis was defined as suspected infection, ≥2 SIRS signs plus one additional sign of organ failure (SBP <90 mmHg, hypoxia (SpO_2_ < 90%), GCS <15 or serum-creatinine >178 mmol/l) [10]. Severe sepsis was thus diagnosed using all domains from Levy et als diagnostic criteria. We used almost all general parameters (except edema and glucoses), one inflammatory marker, one hemodynamic market (systolic blood pressure), one organ dysfunction parameter (creatinine) and Glasgow coma scale as a proxy for perfusion parameters in order to define severe sepsis [10]. qSOFA ≥2 was defined as meeting two or more of these criteria: RR ≥22/min, SBP <100 mmHg or altered mentation, defined as reduction in GCS (GCS <15) [2]. GCS was not registered in patients with dementia or mental retardation [11]. qSOFA was calculated retrospectively based on patients records. Figure 1 displays the different risk stratification tools and how they are used to identify sepsis in clinical practice.

#### Mortality

Electronic hospital records in Norway are updated with mortality data from the Norwegian population registry by using the 11-digit unique identification number of all Norwegian citizens, so that mortality data after discharge from hospital can be reliably assessed, http://www.ssb.no.

### Statistics

We calculated the point estimate and 95% confidence interval (CI) for sensitivity, specificity, and positive (PPV) and negative (NPV) predictive values of the SIRS, SIRS no leuko (≥2 SIRS criteria without leukocytes), qSOFA, and RETTS to identify severe sepsis and predict 7-day and 30-day mortality. The area under the receiving operating characteristic curve (ROC) was used to compare algorithm discrimination. In addition, age- and sex-adjusted associations of severe sepsis, qSOFA, and RETTS with 7- and 30-day mortality were estimated using logistic regression analyses. Individuals, who did not fulfil the qSOFA ≥ 2, severe sepsis RETTS-R or RETTS-O criteria respectively, were used as reference groups. We calculated the sensitivity, specificity, PPV and NPV separately for people aged < and >80 years. Additionally, we investigated the probability of missing values of clinical and laboratory data by complete data (age, sex and triage status). We used multiple imputation, (MI) with chained equation (MICE), known for fully conditional specification of each variable type and used sex, age and triage as regular variables [12], as using all available information including the outcome is preferred in MI [13] The probability of missing values was small (<3% for all measured data and <8% for all constructed variables) and we imputed the missing data 10 times. We compared the ROC and logistic regression analysis after MI with the results from the complete-case analysis. Data were analysed using Stata version 13 (Stata Corp LP, College Station, Texas).

## Results

**Fig. 2:**
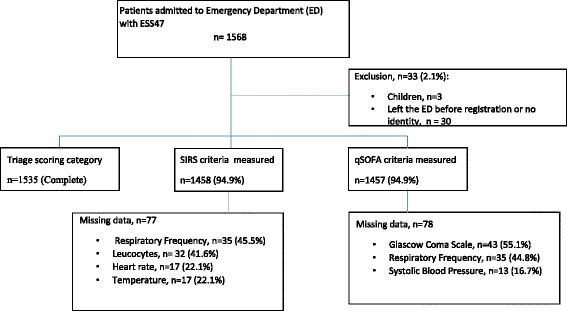
Patient recruitment and information on missing data by severity of illness scoring system in ED

During the study period, 1535 adults were admitted with suspected infection. All patients were triaged according to RETTS, however a small proportion of clinical data for calculation of the SIRS criteria and qSOFA were missing (See Fig. 2). The ≥2SIRS group was younger and closer to the normal range of vital signs upon arrival than the qSOFA and red triage groups (See Tables 1 and 2 for details). Of the 1535 patients admitted with ESS47, 662 patients had sepsis and 108 had severe sepsis (16.3%). 17 (2.6%) patients with sepsis died within 7 days and 42 (6.3%) within 30 days. 8 (7.4%) patients with severe sepsis died within 7 days and 19 (17.6%) died within 30 days. We examined all patient discharge records to those who died within 30 days and found that 60 patients (88%) had sepsis. All patients suffered from serious conditions such as malignant or cardiopulmonary disease or dementia. Among patients with severe sepsis, 37 (34.2%) presented with a diagnosis or vital signs that triggered red alert, and 33 (30.6%) fulfilled the qSOFA ≥ 2 criteria. Of the 1535 patients, 26 (1.7%) died within 7 days and only four (15.4%) of them were identified by the qSOFA ≥ 2 in the ED compared to 17 (65.4%) for SIRS ≥2 and 16 (61.5%) for ≥ orange triage. Sixty-eight (4.4%) patients died within 30 days of admission and the qSOFA upon arrival at the ED identified only 8 (11.9%) compared to 42 (61.8%) for SIRS ≥ 2 and 45 (66.1%) for ≥ orange triage. Sensitivity, specificity, NPV and PPV for all outcomes using the different identification tools are presented in Tables 3, 4 and 5.

**Table 1:** Baseline characteristics by qSOFA and SIRS status

Variable	Total population	*n* (% of Total population)	SIRS ≥2	qSOFA ≥ 2
			*n* = 662	*n* = 59
	*n* (% missing)		*n* (% of category)	*n* (% of category)
Male sex	1535 (0%)	813 (53.0)	348 (52.6)	28 (47.5)
Age category	1535 (0%)			
< 70		994 (64.7)	422 (63.7)	28 (47.5)
70-79		187 (12.2)	90 (13.6)	13 (22.0)
≥ 80		354 (23.1)	150 (22.7)	18 (30.5)
Triage code	1535 (0%)			
Green		146 (9.5)	25 (3.8)	0 (0.0)
Yellow		671 (43.7)	177 (26.7)	7 (11.9)
Orange		609 (39.7)	368 (55.6)	34 (57.6)
Red		109 (7.1)	92 (13.9)	18 (30.5)
Glasgow Coma Scale <15	1492 (2.8)	54 (3.6)	26 (4.1)	29 (49.2)
		Median (IQR)^a^	Median (IQR)	Median (IQR)
Age (years)	1535 (0.0)	62 (41-78)	61 (41-77)	66 (41-81)
Respiratory Rate (min-1)	1500 (2.2)	20 (16-24)	24 (20-26)	24 (2-28)
Heart rate (min-1)	1518 (1.1)	87 (76-100)	100 89-110)	94 (80-108)
Temperature (°C)	1518 (1.1)	37.2 (36.2-38.0)	38.0 (37.1-38.6)	37.4 (36.8
Systolic Blood Pressure (mmHg)	1522 (0.8)	132 (118-147)	130 (116-144)	116 (97-135)
Creatinine (mmol/L)	1491 (2.8)	74 (59-94)	75 61-98)	76 (61-100)
Saturation (SpO_2_ %)	1492 (2.8)	97 (95-99)	97 (95-99)	96 (94-99)

^a^ *IQR* Interquartile range (25-75% percentile)

**Table 2:** Baseline characteristics by triage code category

Variable	*n* (% missing)	Green	Yellow	Orange	Red
		*n* = 146	*n* = 671	*n* = 609	*n* = 109
		*n* (% of category)	*n* (% of category)	*n* (% of category)	*n* (% of category)
Male sex	1535 (0%)	79 (54.1)	343 (51.1)	330 (54.2)	61 (56.0)
Age category	1535 (0%)				
< 70		80 (54.8)	458 (68.2)	397 (65.2)	59 (54.1)
70-79		25 (17.1)	65 (9.7)	76 (12.5)	21 (19.3)
≥ 80		41 (28.1)	148 (22.1)	136 (22.3)	29 (26.6)
Glasgow Coma Scale <15	1492 (2.8)	5 (3.6)	17 (2.6)	20 (3.4)	12 (12.0)
		Median (IQR)	Median (IQR)	Median (IQR)	Median (IQR)
Age (years)	1535 (0.0)	67 (39-80)	59 (38-76)	62 (44-77)	66 (43-80)
Respiratory Rate (min-1)	1500 (2.2)	16 (15-20)	18 (16-20)	20 (18-24)	30 (24-36)
Heart Rate (min-1)	1518 (1.1)	81 (72-88)	83 (73-94)	93 (80-105)	105 (90-122)
Temperature (°C)	1518 (1.1)	105 (90-122)	37.1 (36.7-37.5)	37.7 (37.0-38.6)	38.2 (37.2-39.1)
Systolic Blood Pressure (mmHg)	1522 (0.8)	135 (125-151)	133 (120-148)	130 (116-143)	128 (107-149)
Creatinine (mmol/L)	1491 (2.8)	74 (62-86)	72 (57-88)	76 (62-103)	86 (62-114)
Saturation (SpO2 %)	1492 (2.8)	98 (96-99)	98 (96-99)	97 (95-99)	95 (90-98)

*IQR* Interquartile range (25-75% percentile)

**Table 3:** Sensitivity, Specificity, and Positive (PPV) and Negative Predictive Values (NPV) for severe sepsis by different identification tools in the Emergency department (*n* = 108 cases of severe sepsis among 1535 patients)

Identification tool	Ability to identify severe sepsis	Sensitivity	Specificity	PPV	NPV
	*n* (% of 108 cases)	(95% CI)	(95% CI)	(95% CI)	(95% CI)
SIRS ≥2 (without leukocytes)	80 (74.1%)	0.74 (0.65-0.82)	0.72 (0.70-0.75)	0.18 (0.16-0.19)	0.97 (0.96-0.98)
qSOFA ≥2^a^	33 (30.6%)	0.32 (0.23-0.42)	0.98 (0.97-0.99)	0.57 (0.45-0.68)	0.95 (0.94-0.96)
Red triage	37 (34.3%)	0.34 (0.25-0.44)	0.95 (0.94-0.96)	0.35 (0.27-0.43)	0.95 (0.94-0.95)
Orange triage	55 (50.9%)	0.51 (0.41-0.61)	0.60 (0.58-0.63)	0.09 (0.07-0.11)	0.94 (0.93-0.95)
≥ Orange triage	92 (85.2%)	0.85 (0.77-0.91)	0.55 (0.52-0.58)	0.13 (0.12-0.14)	0.98 (0.97-0.99)

^a^The ability to identify sepsis in % is calculated based on 108 cases of sepsis in the total population, whilst the sensitivity analysis is based on the 103 cases with complete score on qSOFA

**Table 4:** Sensitivity, Specificity, and Positive (PPV) and Negative Predictive Values (NPV) for 7-day mortality by different stratification tools in the Emergency Department (*n* = 26 cases of deaths within 7 days among 1535 patients)

Stratification tool	Ability to identify those who died within 7 days	Sensitivity	Specificity	PPV	NPV
	*n* (% of 26 cases)	(95% CI)	(95% CI)	(95% CI)	(95% CI)
Severe sepsis	8 (30.8%)	0.31 (0.14-0.52)	0.93 (0.92-0.94)	0.07 (0.04-0.12)	0.98 (0.98-0.98)
SIRS ≥2	17 (65.4%)	0.65 (0.44-0.82)	0.55 (0.52-0.57)	0.03 (0.02-0.03)	0.99 (0.98-0.99)
SIRS ≥2 (without leukocytes)	15 (57.7%)	0.58 (0.36-0.76)	0.70 (0.67-0.72)	0.03 (0.02-0.04)	0.99 (0.98-0.99)
qSOFA ≥2	4 (15.4%)	0.16 (0.05-0.36)	0.96 (0.95-0.97)	0.07 (0.03-0.15)	0.98 (0.98-0.99)
Red triage	8 (30.8%)	0.31 (0.14-0.51)	0.93 (0.91-0.95)	0.07 (0.04-0.12)	0.99 (0.98-0.99)
Orange triage	8 (30.8%)	0.31 (0.14-0.52)	0.60 (0.58-0.63)	0.01 (0.00-0.02)	0.98 (0.98-0.99)
≥ Orange triage	16 (61.5%)	0.62 (0.41-0.80)	0.53 (0.51-0.56)	0.02 (0.01-0.03)	0.99 (0.98-0.99)

**Table 5:** Sensitivity, Specificity, and Positive (PPV) and Negative Predictive Values (NPV) for 30-day mortality by different stratification tools in the Emergency Department (*n* = 68 cases of deaths within 30 days among 1535 patients)

Stratification tool	Ability to identify those who died	Sensitivity	Specificity	PPV	NPV
	*n* (% of 68 cases)	(95% CI)	(95% CI)	(95% CI)	(95% CI)
Severe sepsis	19 (27.9%)	0.29 (0.18-0.41)	0.94 (0.92-0.95)	0.18 (0.12-0.24)	0.96 (0.95-0.97)
SIRS ≥ 2	42 (61.8%)	0.64 (0.51-0.75)	0.55 (0.53-0.58)	0.06 (0.05-0.07)	0.97 (0.96-0.98)
SIRS ≥ 2 (without leucocytes)	32 (45.6%)	0.48 (0.36-0.61)	0.70 (0.68-0.72)	0.07 (0.05-0.08)	0.97 (0.96-0.97)
qSOFA ≥2	8 (11.9%)	0.13 (0.05-0.25)	0.96 (0.95-0.97)	0.14 (0.07-0.23)	0.96 (0.96-0.96)
Red triage	14 (20.2%)	0.21 (0.12-0.32)	0.94 (0.92-0.95)	0.13 (0.08-0.19)	0.96 (0.96-0.96)
Orange triage	31 (45.6%)	0.46 (0.22-0.58)	0.61 (0.58-0.63)	0.05 (0.04-0.07)	0.96 (0.95-0.97)
≥ Orange triage	45 (66.1%)	0.66 (0.54-0.77)	0.54 (0.52-0.57)	0.06 (0.05-0.07)	0.97 (0.96-0.97)

In the multivariable regression analyses, the odds ratio (OR) for severe sepsis was higher in the qSOFA ≥ 2 category (24.4, 95% CI 13.243.2) compared with the red triage group (9.7, 95% CI 6.115.5). Among the different identification tools, red triage and severe sepsis had the highest odds ratios for 7-day and 30-mortality, respectively (Additional file 1: Table S1).

In the analysis stratified by age categories <80 versus ≥80, the point estimate for PPV was better for the oldest patient group than for those under 80 years of age for all risk stratification tools in order to identify sepsis, however the statistical uncertainty was large demonstrated with wide and overlapping CIs due to small numbers in each group (data not shown). The probability of missing values on the GCS increased with age (*p* = 0.013) and more severe triage category (*p* = 0.004), whilst the youngest age categories had a borderline larger probability for missing values on vital signs like RR, SBP, temperature and HR (*p* = 0.12). See Additional file 2: Table S2 for details of missing values by age category and triage codes). However, the qSOFA did not perform better in the ROC analysis after MI than in complete case, CC, analysis (see Additional file 3: Table S3) and the results in the logistic regression were also almost identical to the CC analysis (data not shown).

## Discussion

In this observational cohort study, qSOFA had poor sensitivity for detecting severe sepsis, 7-day and 30-day mortality in patients admitted with infection to an ED. The sensitivity was poorer than other risk stratification tools already in use at the study site, RETTS-triage and the SIRS criteria. Thus, our study confirms that the qSOFA fails to be an accurate diagnostic instrument for sepsis upon arrival in the ED when the patients are admitted to the ED with infection.

Sepsis requires urgent identification and every hour of delayed treatment represents increasing mortality [14]. In order to achieve timely treatment, a sensitive screening tool is more important than a specific one [3]. The aim of this study was to do a validation of the clinical usefulness of qSOFA score in assessment of patients at admission for sepsis. Specificity and sensitivity are often used for validation and as a performance criteria for prediction models. The sensitivity and specificity of a decision rule is not only influenced by the quality of the prediction model, but will reveal the effectiveness of the decision rule in clinical practice [15]. In our study, the new qSOFA failed validation as a clinical screening tool with only 32% sensitivity for identifying patients at time of arrival to an ED with severe sepsis. Three studies that previously validated the qSOFA outside the ICU setting with prospective methods supports our conclusion: low sensitivity in identifying septic patients was found in the prehospital setting [16], in the study by Churpek et al., only 9% of the 30,667 patients admitted to an ED or a ward with defined infection suspicion had a qSOFA ≥2 at time of infection suspicion [5] and the qSOFA only had 29.9% sensitivity for detecting organ dysfunction according to the sepsis 3 definition in an Australian ED [6]. The third study, which used expert groups and worst qSOFA score during the stay in the ED, found that qSOFA performed better than the SIRS criteria [7]. However, two objections remain; Firstly, the qSOFA can vary over a short period of time, and ED’s needs tools to detect sepsis at the time of the arrival and a triage tool like RETTS seems better than q-sofa for this purpose; Secondly, in most ED’s the patient is not met by sepsis experts, but interns, thus they need a triage tool that can be used by nurses and general physicians and RETTS triage seems better for this purpose as well. After this study, in order to raise patient safety, the ED is strengthened with two Senior Resident Attending Physician to raise the expertise in assessing critical illness, and the qSOFA might perform better at sites with such recourses in place. The qSOFA score was not only designed to be a screening tool for severe sepsis, but also as a risk stratification tool in order to find those patients that are likely to fare poorly [2]. Recently, Churpek et al. found that the qSOFA performed poorer than the NEWS and the MEWS for predicting in-hospital mortality and ICU transfer in non-ICU patients [5]. We add information on the RETTS triage system, which like the NEWS, measures seven signs of physical deterioration (RR, HR, BP; Temperature, mental function, saturation and supplemental oxygen) instead of only three vital signs in the qSOFA score (RR, BP, GCS). A RETTS ≥ -orange response will compare to a NEWS >3 [9, 17]. Since mortality is measured in slightly different ways in our and the study by Churpek et al. [5], it is not easy to directly compare the sensitivity for NEWS, 72% for in-hospital mortality, with 61% sensitivity for 7-day mortality and 66% for 30-day mortality for RETTS ≥ orange alert. Thus, our study adds information on the RETTS triage system, the two highest acuity levels together had a high sensitivity (85%) for identifying sepsis. The RETTS red and orange response triage ensures quick doctoral response and thus may be a useful screening tool.

Further, we showed that a SIRS-score ≥2 also had higher sensitivity than qSOFA in predicting both 7-day and 30-day mortality. The identification and treatment of sepsis is challenging, since this is a heterogeneous group, in terms of age, comorbidities and type of infection [18]. A study that argued against the SIRS criteria as an identification tool found nevertheless that the SIRS-criteria ≥2 had high sensitivity (88%) for identifying patients with infection and organ failure [19].

One of the strongest arguments for the new qSOFA score was that it was a quick and easy bedside tool for the identification of sepsis [2]. The missing values on the GCS in our study depended on high age and high acuity levels and this indicates that GCS is not an easy bedside tool for these vulnerable patients, nor is the determination of altered mental status in patients with dementia [11]. In our ED, the GCS is not assessed on persons with dementia which can explain the opposite conclusion compared to Freund et al. [7] that solved the problems with qSOFA with replacing the scale with the presence of an altered mental status. Previously, the GCS item has been reported to be problematic as a criterion in sepsis decision making for patients with stroke, encephalitis, intoxication, hypoxemia and hypercapnia or patients that received procedural analgesics. GCS was missing most among the oldest patients, especially in patients with dementia, and in the patients with highest acuity levels. This indicates that assessing GCS is not an easy task in these patients. Alternatively, lactate could be of value in sepsis patients in which GCS is difficult to assess as lactate is an indicator for hypoperfusion that is strongly correlated with sepsis. Our study shows that if we used the SIRS criteria ≥ 2 without leukocytes it still out-performed the qSOFA. Accordingly, the SIRS criteria are as quick as the qSOFA, without the ambiguity related to the GCS score.

While there is a trigger to perform an ECG in all patients with chest pain for early recognition of myocardial infarction, the new sepsis consensus suggests that the trigger for sepsis surveillance should be evidence of dysfunction in ≥ 2 organ systems [2]. It should therefore be no surprise that clinicians are worried that the qSOFA criteria seems to identify the patient too late in the course [3, 20], in-fact up to 12 h later than for the SIRS criteria [5]. A recent study from Torsvik et al. [21] showed that education of ward staff in the continuation of systematic SIRS and organ failure-triage (SOF-triage) might prevent patients with blood stream infection (BSI) from progressing to life-threatening sepsis. This system might be one solution for the interim patients that are suspected to have sepsis even if they do not meet the qSOFA score ≥ 2 criteria [21]. Interestingly, the SOF-triage cut off for starting sepsis treatment is comparable to a NEWS score of 3 which is recommended by the Royal College of Emergency Medicine for escalating treatment of patients with suspected infection [21–23].

### Strengths and limitations

Trained triage nurses assessed all the patients at arrival. The nurses might have missed some patients with infection at the triage, i.e. given them another RETTS-diagnosis, like ESS53, which is an unspecific triage code. However, even this potential miss-classification could affect late detection of sepsis in some patients, it would not affect the clinical usefulness of the screening tools for the patients that did enrol in the study.

Further, not all parameters from Levys et als diagnostic sepsis criteria from 2003 was collected at arrival in ED, which could also have led to an underdiagnosis of sepsis. However, not any of the screening tools, neither RETTS, SIRS nor qSOFA utilize the data we missed in our data collection. Thus, these screening tools would not have found more patients with sepsis even if we had this information. In this study, we could compare the performance of commonly used prediction tools for severe sepsis and mortality in patients with infection in a quite large prospective observational study. Information on the variables included in the prediction tools was almost complete, and the results in the multiple imputation analysis were in line with the complete case analysis. As all information included in the prediction tools was recorded while the patient was in the ED, it was not influenced by later changes in the patients’ clinical status. One limitation is the lack of information on comorbidities that may be important in judging the usefulness of trigger systems for detecting sepsis and mortality related to sepsis. Our study is likely representative for Norwegian hospitals with local and regional responsibilities for sepsis treatment. We cannot exclude that the performance of the qSOFA may be different in other study populations; nonetheless, our results agree with those of studies in the prehospital setting, EDs in UK and Australia showing a poor performance of the qSOFA [5, 6].

## Conclusion

In this observational cohort study, qSOFA failed to detect two thirds of severe sepsis cases among patients admitted to an ED with suspicion of infection. Further, the qSOFA failed to be a risk stratification tool as the sensitivity to predict 7-day and 30-day mortality was low. The sensitivity was poorer than other warning scores already in use at the study site, RETTS-triage and the SIRS criteria. Since the ED not only should identify those who are critically ill of sepsis at time of arrival, but also represent an important identification point for those patients that are likely to become so, we cannot recommend ED’s that already has triage systems in place to implement the qSOFA.

## Additional files

Additional file 1: Table S1. Odds Ratios for severe sepsis and 7- and 30-day mortality for the different stratification tools. (DOCX 13 kb)

Additional file 2: Table S2. Detailed information on missing values bye age group and triage category. (DOCX 12 kb)

Additional file 3: Table S3. ROC Area for 7 and 30 days mortality in Complete Case and Multiple Imputation analysis. (DOCX 23 kb)

## Abbreviations

ED: Emergency Department
ESS 47: Emergency symptoms and signs algorithm for infection
GCS: Glasgow coma scale
HR: Heart rate
NEWS: National early warning score
qSOFA: quick sequential related organ failure assessment
RETTS: Rapid emergency triage and treatment system
RR: Respiratory rate
SBP: Systolic blood pressure
SIRS: Systemic Inflammatory response syndrome
