# Supplementary material for: Poor performance of quick-SOFA (qSOFA) score in predicting severe sepsis and mortality – a prospective study of patients admitted with infection to the emergency department
Source: Scand J Trauma Resusc Emerg Med. 2017 Jun 9;25:56. doi: 10.1186/s13049-017-0399-4 (PMC5466747; doi:10.1186/s13049-017-0399-4)
Supplement: Supplementary file 1 — Odds Ratios for severe sepsis and 7- and 30-day mortality for the different stratification tools. (DOCX 13 kb) [file 13049_2017_399_MOESM1_ESM.docx]

| Additional file 1: Table S1 Odds Ratios for severe sepsis and 7- and 30-day mortality for the different stratification tools | | | |
| --- | --- | --- | --- |
| Outcome | Crude model | Age- adjustment | Age- and Sex- adjustment |
| **Severe sepsis** |  |  |  |
| qSOFA≥2 | 24.4 (13.2-43.2) | 23.6 (13.2-42.2) | 23.6 (13.2-42.2) |
| Red triage | 9.7 (6.1-15.4) | 9.3 (5.8-14.3) | 9.3 (5.8-14.9) |
| ≥Orange triage | 7.1 (4.1-12.2) | 7.0 (4.1-12.1) | 7.0 (4.1-12.1) |
| **Death within 7 days** |  |  |  |
| severe sepsis | 5.9 (2.5-14.0) | 4.3 (1.8-10.3) | 4.2 (1.7-10.1) |
| qSOFA≥2 | 4.8 (1.6-14.4) | 3.7 (1.2-11.7) | 3.7 (1.2-11.6) |
| Red triage | 6.2 (2.6-14.6) | 5.1 (2.1-12.4) | 5.1 (2.1 - 12.3) |
| ≥Orange triage | 1.8 (0.8-4.1) | 1.8 (0.8-4.1) | 1.8 (0.7-3.9) |
| **Death within 30 days** |  |  |  |
| severe sepsis | 5.9 (3.3-10.3) | 4.6 (2.5-8.5) | 4.5 (2.4-8.4) |
| qSOFA≥2 | 4.1 (2.8-9.0) | 3.3 (1.4-7.9) | 3.3 (1.4-7.9) |
| Red triage | 3.2 (2.0-7.0) | 3.2 (1.6-6.2) | 3.1 (1.5-6.1) |
| ≥Orange triage | 2.3 (1.4-3.9) | 2.4 (1.4-4.1) | 2.3 (1.2-3.8) |
| ***In the logistic regression analyses, patients who did not have a q-SOFA score ≥2, red triage, orange triage or severe sepsis respectively, were used as reference groups. | | | |
|  |  |  |  |
