# Supplementary material for: Poor performance of quick-SOFA (qSOFA) score in predicting severe sepsis and mortality – a prospective study of patients admitted with infection to the emergency department
Source: Scand J Trauma Resusc Emerg Med. 2017 Jun 9;25:56. doi: 10.1186/s13049-017-0399-4 (PMC5466747; doi:10.1186/s13049-017-0399-4)
Supplement: Supplementary file 2 — Detailed information on missing values bye age group and triage category. (DOCX 12 kb) [file 13049_2017_399_MOESM2_ESM.docx]

| Additional file 2: Table S2. Detailed information on missing values bye age group and triage category. | | | |
| --- | --- | --- | --- |
| Categories | n missing | proportion missing  (of row total) | p between categories |
| *Age* |  |  | 0.013 |
| Age<70 (n=994) | 19 | 1.9 |  |
| Age 70-80 (n=187) | 7 | 3,7 |  |
| Age>80 (n=354) | 17 | 4,8 |  |
| *Triage* |  |  | 0.004 |
| Green (n=146) | 5 | 3,4 |  |
| Yellow (n=671) | 16 | 2,4 |  |
| Orange (n=609) | 13 | 2,1 |  |
| Red (n=109) | 9 | 8,3 |  |
