# Supplementary material for: Poor performance of quick-SOFA (qSOFA) score in predicting severe sepsis and mortality – a prospective study of patients admitted with infection to the emergency department
Source: Scand J Trauma Resusc Emerg Med. 2017 Jun 9;25:56. doi: 10.1186/s13049-017-0399-4 (PMC5466747; doi:10.1186/s13049-017-0399-4)
Supplement: Supplementary file 3 — ROC Area for 7 and 30 days mortality in Complete Case and Multiple Imputation analysis. (DOCX 23 kb) [file 13049_2017_399_MOESM3_ESM.docx]

| Additional file 3: Table S3 ROC Area for 7 and 30 days mortality in Complete Case and Multiple Imputation analysis. | | |
| --- | --- | --- |
|  | ROC Area 7-days mortality | ROC Area 30-days mortality |
| Complete Case |  |  |
| Multiple Imputation |  |  |
